# Mapping Strategies and Interventions to Reach Zero-Dose and Under-Immunized Children: A Scoping Review

**DOI:** 10.3390/vaccines14100848

**Published:** 2026-09-26

**Authors:** Tshepiso Mbangiwa, Imen Ayouni, Susanne Noll, Dilshaad Brey, Heidi Reynolds, Edina Amponsah-Dacosta, Benjamin M. Kagina

**Affiliations:** 1 Vaccines for Africa Initiative & NITAG Support Hub, School of Public Health, Faculty of Health Sciences, University of Cape Town, Cape Town 7925, South Africa; aynime001@myuct.ac.za (I.A.); susanne.noll@uct.ac.za (S.N.); dilshaad.brey@uct.ac.za (D.B.); edina.amponsah-dacosta@uct.ac.za (E.A.-D.); benjamin.kagina@uct.ac.za (B.M.K.); 2 Gavi, the Vaccine Alliance, Chemin du Pommier 40, Le Grand Saconnex, 1218 Geneva, Switzerland; hwreynolds@gmail.com; 3 Institute of Infectious Disease and Molecular Medicine (IDM), Faculty of Health Sciences, University of Cape Town, Cape Town 7925, South Africa

**Keywords:** zero-dose (ZD), under-immunized, childhood vaccination, hard-to-reach communities, interventions and strategies

## Abstract

Background: Zero-dose (ZD) children, classified as those who have not received their first dose of diphtheria–tetanus–pertussis vaccine, remain at high risk of vaccine-preventable diseases globally. Despite progress, an estimated 14.3 million children were ZD in 2024. We systematically mapped global evidence on interventions to reach ZD and under-immunized children to inform policy and planning towards vaccine equity. Methods: We conducted a scoping review of peer-reviewed studies published between 2015 and 2025 reporting interventions to reach ZD or under-immunized children under five years. We searched PubMed, Scopus, Web of Science, and EBSCOhost databases. Two reviewers independently extracted data using standardized forms. We analyzed interventions thematically and by World Bank income classification. Results: Of the 37 included studies, 24% (*n* = 9) originated from low-income countries (LICs), 65% (*n* = 24) from lower-middle-income countries, 5% (*n* = 2) from upper-middle-income countries, and 5% (*n* = 2) from high-income countries. Mobile outreach programmes were the most prevalent intervention at 46% (17/37), followed by engagement with religious and community leaders at 41% (15/37) and community healthcare worker involvement at 27% (10/37). We identified eight thematic intervention areas: community engagement, community and health workforce, health systems strengthening, outreach and service delivery, technology-driven approaches, monetary incentives, integrated human–animal campaigns, and leveraging military and security forces. Intervention patterns varied by income level, with LICs emphasizing community engagement and mobile outreach, while higher-income countries utilized technology and incentive-based approaches. Five studies specifically reported on ZD children, with most reporting improvements in first dose of DTP/Penta vaccine uptake following intervention implementation. Conclusions: Multi-faceted, context-adapted interventions show promise for reaching ZD children. However, clearer definitional standards are needed to distinguish ZD from under-immunized populations. Strategies identified in this review can guide policymakers in designing and prioritizing further evaluation of interventions to achieve equity in immunization coverage globally.

## 1. Introduction

Vaccination in childhood is a cornerstone of global health efforts to control, eliminate and eradicate vaccine-preventable diseases such as whooping cough, tuberculosis, polio, and measles. Recognized as one of the most effective public health interventions, childhood immunization averts millions of deaths annually [1]. However, despite concerted global efforts to ensure availability of essential childhood vaccines, an alarming number of children remain unvaccinated or under-immunized [2]. A 2024 report by WHO estimates that approximately 14.3 million children worldwide were “zero-dose”, children who missed their first diphtheria, tetanus, and pertussis (DTP) vaccine dose, a setback exacerbated by disruptions caused by the COVID-19 pandemic [3].

We recognize there is no internationally agreed one definition of ZD children. As an example, there is a study that proposes three definitions of ZD in children of 12–23 months; (1) Penta-ZD children are those who have not received DTP1; (2)Truly ZD children refer to those who have not received either Bacillus Calmette–Guérin (BCG), polio (OPV0), DTP1 or measles-containing vaccines, and finally, (3) misclassified ZD children refer to those who have not received DTP1 but have received at least BCG, OPV0 or measles-containing vaccines [4]. Several studies have reported on ZD children using the non-receipt of OPV0, which is especially common in polio eradication campaigns [5,6,7].

In this study, ZD children are defined using the GAVI and WHO definitions as children who have not received the first dose of diphtheria, pertussis, and tetanus (DTP1) or pentavalent1 (Penta1) or hexavalent vaccine. On the other hand, under-immunized children are defined as children who have received some doses but have missed one or more of the recommended vaccines required for full immunization or have not taken the third dose of DTP (DTP3)-containing vaccine; however, this is subject to availability within the context of their countries [1,2]. The burden of ZD (unvaccinated) and under-immunized (under-vaccinated) children is disproportionately concentrated in low- and middle-income countries (LMICs), where systemic barriers hinder access to vaccines.

To address these disparities, Gavi, the vaccine alliance, launched its Gavi 5.0 strategy in 2019, adopting the guiding principle of “leaving no one behind”. Central to this strategy is the reduction in ZD children by 25% by 2025 and by 50% by 2030, in alignment with the United Nations’ Sustainable Development Goals (SDGs) for 2030 [2] and the Global Immunization Agenda 2030 (IA2030). Gavi introduced the Identify–Reach–Measure–Monitor–Advocate (IRMMA) framework, a comprehensive approach emphasizing the need to identify and reach ZD communities through tailored and targeted interventions, measure and monitor progress, and advocate for policy and resource mobilization [8].

In settings prevalent with ZD children, evidence shows these children are disproportionately living in urban slums, remote rural areas, and conflict-affected regions, where complex barriers including poverty, misinformation, weak health systems, vaccine hesitancy, and migration impede immunization efforts [9,10]. Studies show that a significant proportion of ZD children live in peri-urban and urban settings, emphasizing the need for strategies tailored to unique urban health challenges [11,12]. The COVID-19 pandemic widened these gaps, as healthcare systems in many LMICs shifted resources toward pandemic-related priorities [13,14].

ZD and under-immunized children are at heightened risk of infectious diseases, which are especially prevalent in LMICs. According to recent estimates, millions of children worldwide remain unvaccinated, with the highest burden concentrated in conflict-affected regions, remote rural areas, and urban slums where access to healthcare services is limited. Social, economic, and systemic barriers including poverty, misinformation, weak health systems, and vaccine hesitancy contribute to the persistence of ZD children, making targeted interventions essential. Evidence suggests that community-based strategies, such as deploying community healthcare workers (CHWs), leveraging digital health technologies, and strengthening immunization supply chains, have shown promise in improving vaccine access.

Although some literature reviews have been conducted on interventions to improve immunization coverage and drivers of ZD, there are still gaps in synthesized evidence specific to reaching ZD children. While Beaulieu et al. provides a comprehensive account of prevalence and barriers to reaching ZD children, they do not report on any interventions or strategies to reach this population, a gap our review sets out to fill [15]. The other existing reviews are limited in scope, often focusing on some regions or countries, intervention types, or specific vaccines, rather than providing a comprehensive global overview of strategies targeting this population [12,15,16]. While ZD children are predominantly found in LMICs, studies indicate that under-immunization remains a concern even in HICs, particularly among marginalized communities [17,18,19]. Without a thorough synthesis of available interventions globally, it remains challenging to determine which approaches are most effective, scalable, and adaptable across different contexts. This scoping review is therefore essential to consolidate available evidence, identify knowledge gaps, and provide insights that can inform future policies and programmes aimed at reducing the number of ZD children and scaling up equitable vaccine access.

Therefore, the aim of this scoping review was to map interventions and strategies aimed at reaching ZD children under 5 years globally, particularly those in hard-to-reach communities with immunization services.

## 2. Materials and Methods

This scoping review was conducted in alignment with the Preferred Reporting Items for Systematic Reviews and Meta-Analyses extension for scoping reviews (PRISMA-ScR) [20]. The protocol for this review has been registered on open science forum (OSF) (https://doi.org/10.17605/OSF.IO/CDB9V, accessed on 6 May 2025).

### 2.1. Literature Search Strategy

Guided by information specialists (SN and DB), the search strategy was designed to identify studies related to infants, children, and neonates concerning immunization and vaccination, with a specific focus on ZD, unvaccinated, under-vaccinated, or hard-to-reach populations, targeting strategies, interventions, and innovations to improve vaccine coverage. Searches were conducted across multiple databases and platforms including PubMed, Scopus (MEDLINE and Embase), Web of Science (Core Collection) and EBSCOhost (MEDLINE, Academic Search Premier, CINAHL, Health Source: Nursing/Academic Edition, Africa-Wide Information, Health Source-Consumer Edition). The search was conducted in May 2025.

By employing a comprehensive and systematic search strategy, we aimed to identify published evidence on strategies, interventions, and innovations targeting ZD, under-immunized, and hard-to-reach children. The search utilized both controlled vocabulary and free-text terms to maximize sensitivity and ensure broad coverage of the relevant literature. The search strategy was structured around three core concepts: population, immunization, and intervention which were combined using Boolean operators to ensure that only studies meeting all three criteria were retained.

### 2.2. PubMed Search Strategy

For the population component, Medical Subject Heading (MeSH) terms including “infant”, “infant newborn”, “child”, “child preschool”, and “pediatrics” were employed to capture studies involving children under five years of age. Immunization-related concepts were identified using MeSH terms such as “immunization”, “vaccination”, and “vaccines”. To specifically target studies addressing vaccine equity and access, the search incorporated title and abstract terms including “ZD”, “zero-dose”, “unvaccinated”, “under-vaccinated”, “non-immunized”, “hard-to-reach”, and “missed communities”. Additionally, intervention-focused terms such as “strategies”, “interventions”, and “innovations” were included to identify the implementation-relevant literature (see Table 1).

### 2.3. Search Strategy

To minimize potential bias from disease-specific vaccination programmes that are not routinely targeted at children, studies focusing exclusively on SARS-CoV-2, human papillomavirus (HPV), and influenza virus were excluded from the search. The temporal scope was restricted to publications from 2015 to 2025. Furthermore, the search was limited to studies involving children under five years of age to maintain focus on the target population.

This structured methodological approach was designed to systematically map the breadth and nature of existing evidence on interventions targeting ZD and under-vaccinated children, identify key thematic domains, and elucidate gaps in implementation strategies across diverse geographical and programmatic contexts.

### 2.4. Inclusion Criteria

The PICOTS (population, intervention, comparator/control, outcomes, timeframe) framework was used to define the inclusion criteria as below:

1. Population: ZD or under-immunized children (<5 years) across global contexts.
2. Intervention: Strategies and interventions aimed at reaching ZD and under-immunized children.
3. Comparison: No direct comparison group; interventions will be analyzed across diverse geographic regions and socio-economic contexts to identify patterns and variations.
4. Outcomes: Increased vaccination coverage among ZD and under-immunized children, improved access to immunization services, and identification of effective, scalable, and context-specific strategies.
5. Study Types: Only available primary studies, including observational and interventional designs, and peer-reviewed and full-text articles were considered with no language restrictions.
6. Timeline: Studies conducted and published between 2015 and 2025; this timeframe aligns with the approval of the Gavi 4.0 strategy which was implemented between 2016 and 2020, which prioritized reaching underserved and unreached populations as part of its core mandate.

### 2.5. Exclusion Criteria

1. Studies focusing on interventions in children over five years or adults, review articles, the grey literature, and modelling studies. Modelling studies were excluded as the aim of this review was to identify and describe strategies and interventions that have been directly implemented and evaluated in real-world settings; mathematical projections of intervention impact were considered outside the scope of this question. Reviews, including systematic reviews and narrative reviews, were excluded to avoid double-counting of primary studies and to ensure that the evidence base reflects the original research. Where relevant reviews were identified during screening, their reference lists were examined to identify eligible primary studies.
2. Grey literature sources were not searched in this review. The rationale behind this decision was that the review’s primary objective was to map peer-reviewed evidence on strategies and interventions for reaching ZD and under-immunized children, identified through academic databases.
3. To maintain relevance to routine childhood immunization programmes, studies related to COVID-19, HPV, and influenza were excluded.
4. Studies carried out before 2015 and after 2025.

### 2.6. Study Screening and Selection Process

All identified articles were exported to EndNote (version 21; Clarivate, Philadelphia, PA, USA) and subsequently imported into Rayyan (web version, Rayyan Systems Inc., Cambridge, MA, USA; accessed February 2025 to January 2026) [21] for screening. Duplicates were removed and abstracts and full-text articles were subsequently screened for eligibility using Rayyan [21]. Following a pilot test, titles and abstracts were screened by two independent reviewers (TM and IA) for assessment against the eligibility criteria. Full texts of studies meeting the inclusion criteria were then assessed to confirm eligibility. A PRISMA-ScR workflow was used to maintain an audit trail of the reasons for exclusion of studies that did not meet the full-text eligibility criteria [20].

### 2.7. Data Extraction, Analysis and Synthesis

Data were systematically extracted from studies included in the scoping review by two independent reviewers (TM and IA) using a google data extraction sheet (web version, Google LLC, Mountain View, CA, USA; accessed May 2025 to April 2026) that had been validated with 5 studies to test whether data being retrieved was useful. The extraction process ensured consistency and minimized bias through the application of pre-defined criteria. Primary study identifiers included author names, country, year of publication, title, study aim, setting, income classification, and Digital Object Identifier (doi) to ensure accurate referencing. Data of interest comprised intervention or strategy type, detailed descriptions of interventions, reported outcomes, and stated limitations.

Detailed summaries of the extracted data were presented in tables and figures, with findings organized under relevant themes and subcategories. Study findings are categorized into distinct thematic areas, contextualized by income classification and whether children were ZD or under-immunized. Where included studies reported non-receipt of OPV0 rather than DTP1/Penta1 as their outcome, typically within polio supplementary immunization activities (SIAs) in conflict-affected or high-polio-risk settings, these were extracted and reported separately from the WHO/Gavi zero-dose indicator and were not classified as either ‘zero-dose’ or ‘truly zero-dose’ per Wonodi and Farrenkopf’s composite definition [4]. These results were presented and discussed accordingly. Gaps in the existing evidence base requiring further research are identified and discussed.

Consistent with the exploratory, evidence-mapping purpose of a scoping review and the guidance of the PRISMA-ScR framework, formal critical appraisal of methodological quality or risk of bias was not conducted [20]. This decision reflected the marked heterogeneity of study designs identified during screening, which ranged from randomized controlled trials to descriptive programme evaluations without comparison groups, precluding consistent application of a single appraisal tool across the included evidence base.

## 3. Results

We retrieved a total of 917 articles, of which 385 were duplicates and subsequently removed. A total of 532 unique records underwent title and abstract screening, resulting in the exclusion of 480 articles that did not meet the inclusion criteria. The remaining 52 full-text articles were screened for eligibility, and 15 were excluded for the following reasons: wrong outcome (*n* = 8), participants aged over 10 years (*n* = 1), inappropriate study design (*n* = 5), and the grey literature (*n* = 1). Consequently, 37 articles met the eligibility criteria and were included in the scoping review (see full list in Supplementary Table S1). The extracted data are presented in a PRISMA chart below (see Figure 1).

### 3.1. Income Classification of Countries Represented Across the Included Studies

Of the 37 studies included in this review, 24% (*n* = 9) originated from low-income countries (LICs), 65% (*n* = 24) from lower-middle-income countries (LMICs), while upper-middle-income countries (UMICs) and high-income countries (HICs) contribute 5% (*n* = 2) and 5% (*n* = 2) of the studies, respectively (see Supplementary Figure S1). This distribution highlights a strong representation of research conducted in LICs and LMICs, which is particularly relevant given that these settings bear the highest burden of ZD and under-immunized children globally. The focus on these countries aligns with the review’s aim to understand and address barriers to vaccination coverage where they are most critical. Although fewer studies come from UMICs and HICs, their inclusion provides valuable insights into innovative strategies and interventions for health system strengthening that could be adapted or scaled to lower-income contexts. Overall, the geographic and economic diversity of the studies included ensures a comprehensive overview of interventions across different health system capacities and socio-economic settings.

### 3.2. Frequency of Interventions and Strategies to Reach ZD or Under-Immunized Children

When analyzing the range of interventions and strategies reported across the included studies, mobile outreach programmes emerged as the most prevalent approach, featured in 46% (17/37) of the studies (Figure 2). This was closely followed by engagement with religious and community leaders, which appeared in 41% (15/37) of the studies, underscoring the importance of trusted local influencers in improving vaccination uptake. Interventions involving CHWs were also significant, reported in 27% (10/37) of the studies, highlighting their critical role in reaching ZD and under-immunized children. Other noteworthy strategies included training programmes for healthcare workers (HCWs) or CHWs, accounting for 16% (8/37) of the interventions, as well as supply chain monitoring at 14% (5/37) to ensure vaccine availability and reduce stockouts. Vaccination campaigns were reported in 11% (4/37) of the studies, often complementing routine services to boost coverage. Collectively, these interventions reflect a multi-faceted approach and 57% (21/37) used more than one intervention/strategy aimed at overcoming both demand- and supply-side barriers to immunization.

### 3.3. Thematic Areas of Strategies and Interventions to Reach ZD and Under-Immunized Children

This scoping review identified eight thematic areas covering interventions and strategies to improve vaccination amongst ZD and under-immunized children (see Supplementary Figure S2) with geographic locations of the studies in Africa and South-East Asia (see Supplementary Figure S3), with some countries represented by more than one study, and intervention use varied notably by income classification (Table 2). These involved the community and health workforce, community engagement, health systems strengthening, outreach and service delivery, monetary incentives, technology, integrated human and animal campaigns and lastly, leveraging of military and security forces.

As summarized in Table 2, the range and complexity of intervention strategies varied considerably across income settings. LICs relied predominantly on foundational approaches including community engagement, CHW and health workforce deployment, outreach and service delivery, and health systems strengthening, with integrated human–animal vaccination campaigns representing a unique equity-focused strategy in pastoralist and nomadic contexts. LMICs built upon this foundation with a broader intervention mix, notably introducing technology-driven tools and the leveraging of military and security forces, reflecting comparatively greater access to digital infrastructure and formal institutional mechanisms. UMICs demonstrated a further shift toward targeted, technology-enabled, and community-centred strategies, with less reliance on broad campaigns (outreach and service delivery). HICs’ approaches included technology and monetary incentives. Taken together, these patterns suggest that intervention complexity and technological integration increase alongside income classification, while community-centred and outreach-based strategies remain relevant across all settings, particularly where health system capacity and geographic accessibility continue to constrain routine immunization delivery.

Among the eight thematic areas identified, community engagement, encompassing caregiver education, social mobilization, and engagement with local and religious leaders, was the most widely reported theme, appearing as a supporting component in multi-component interventions across 14 countries including Nigeria, Pakistan, Afghanistan, and Ethiopia, though it was not evaluated as a standalone strategy (Table 3).

Closely linked to this was the deployment and training of community healthcare workers and healthcare workers (CHWs & HCWs), which emerged as a core delivery strategy across fragile, rural, and hard-to-reach settings; while Uganda reported CHW-led approaches as a standalone intervention, the theme was more commonly embedded within broader packages across Angola, Cameroon Nigeria, Ethiopia, Somalia, Afghanistan, Pakistan, Chad and Cote d’Ivoire.

Outreach and service delivery, including mobile clinics, periodic intensification of routine immunization, and supplementary immunization activities, were similarly prominent, functioning as a component of combined strategies in Angola, Nigeria, Cameroon, Pakistan, Somalia and Ethiopia.

Technology-driven interventions, including mobile health applications, GIS mapping, SMS reminders, and digital tracking tools, were reported across a wide range of settings from urban to nomadic, with Pakistan, Bangladesh, and Denmark implementing these as standalone approaches, while many other countries integrated them within broader intervention packages.

Health systems strengthening (HSS) was reported primarily as part of multi-component packages in Angola, Cameroon, Nigeria, Ethiopia, Chad and Pakistan, and was notably multi-faceted in its scope. Rather than addressing a single bottleneck, HSS interventions operated across several interconnected domains simultaneously, encompassing improvements to vaccine supply chain management, cold chain infrastructure, workforce capacity building, governance and accountability mechanisms, and integration of immunization services within broader primary healthcare delivery. This systemic approach reflects the recognition that barriers to reaching ZD children are rarely attributable to a single cause but instead arise from cumulative failures across multiple levels of the health systems. As such, HSS was commonly implemented alongside mobile outreaches, CHW deployment, and technology-driven tools, functioning as the structural foundation upon which other intervention components could operate effectively and sustainably.

The remaining three thematic areas were more contextually specific. Leveraging of military and security forces was applied exclusively in conflict-affected settings, notably in Nigeria and Angola, to facilitate access to previously unreachable populations. Integrated animal and human vaccination campaigns, grounded in a One Health approach, were tailored to pastoralist and nomadic communities in Somalia and Chad. Finally, monetary incentives were represented solely by Australia’s “No Pay, No Jab” policy, the only high-income-country strategy identified, demonstrating that demand-side financial levers can also drive improvements in vaccination coverage at the national levels.

### 3.4. Reported Changes in Vaccine Coverage Following Multi-Faceted Interventions in Low-Resource and Hard-to-Reach Settings

Most studies included in this review reported improvements in vaccine coverage following the introduction of various interventions, although statistical significance was not consistently assessed or reported across studies. The duration of follow-up varied across studies, with most measuring outcomes within one year of intervention implementation and a few assessing outcomes beyond one year (long-term), as presented in Table 3.

For example, a study conducted in a rural, hard-to-reach area of Afghanistan, a low-income country (LIC), employed multiple strategies including engagement of religious and community leaders, communication campaigns, educational interventions, and activities led by healthcare workers (HCWs) including community healthcare workers (CHWs) [39]. This study used a pre–post design with a comparison district, and outcomes were measured at 12 months post-intervention. Hemat et al. reported a significant increase in full immunization coverage in the intervention district, rising from 40.0% at baseline to 78.6% at follow-up (*p* < 0.001). Among children aged 24–35 months, full or partial immunization coverage increased by 20.6 percentage points, from 66.1% to 86.7% (*p* < 0.001). Significant improvements were also noted in antigen-specific coverage, with increases of 20.2, 45.2, 54.4, 51.6, and 42.4 percentage points for Bacille Calmette–Guérin (BCG), Penta1, Penta-2, Penta-3, and Measles-1 vaccines, respectively [39].

Similarly, Kaduru et al., conducted a study in a hard-to-reach and security-compromised region of Nigeria, using a quasi-experimental design over 18 months without a comparison group, with outcomes assessed at six months into the intervention period and six months post-intervention [24]. The intervention employed community theatre as a demand-generation strategy, whereby community members, particularly caregivers, were engaged as cast members and received training in both vaccination messaging and theatrical performance. Performances incorporated traditional songs, local languages, and emotionally resonant themes including awe, pride, love, and humour to foster behaviour change in a culturally meaningful way. Plays were staged at community and religious gatherings and were followed by structured discussions led by community healthcare workers to reinforce and clarify key immunization messages. By leveraging a familiar, participatory, and accessible medium, the approach aimed to address vaccine hesitancy, dispel misconceptions, and strengthen community awareness and acceptance of immunization services in a setting where conventional health communication strategies may have had limited reach. The proportion of fully immunized children rose from 46% at baseline to 55% at midline, and further to 84% post-intervention, representing a 38 percent increase over the study period. Additionally, child health cards were observed in 65%, 70%, and 75% of the households at baseline, midline, and endline surveys, respectively, providing a measure of verification of immunization status [24].

In Northern Thailand, Kaewkungwal et al. showed that mobile phone applications could improve vaccine coverage among highland minority and stateless populations using pre/post assessments with outcomes measured at 6 months and one year post-intervention [35]. The applications were designed to support healthcare workers in tracking vaccination schedules, identifying children due or overdue for immunization, and facilitating timely follow-up within hard-to-reach highland communities. Modest improvements in vaccine coverage were observed among children aged one year, with OPV and DTP coverage increasing from 91.7% to 94.4%, and measles vaccination rising from 89.2% to 89.6%; *p*-values for these comparisons were not reported. The introduction of tablet applications coincided with positive trends in monthly coverage: among one-year-olds, the monthly OPV-DTP coverage range improved from 81 to 98% pre-intervention to 86 to 100% post-intervention, and monthly measles coverage improved from 73 to 97% to 83 to 97%. Among two-year-olds, OPV-DTP coverage rose marginally from 86.3% to 86.6%, and among four-year-olds, coverage improved from 73.2% to 78.0%, with monthly OPV-DTP coverage shifting from a range of 54 to 87% to 58 to 89% [35].

**Studies Reporting Improvements in ZD (no DTP1/Penta1) and OPV0 Coverage following Interventions**.

Five studies reported on ZD children and five on children who had not received OPV0 (one study reported on both ZD and OPV0 children); improvements in vaccine uptake were observed for both DTP1/Penta1 and OPV0 following intervention implementation. For Penta1 coverage, Hemat et al. reported the largest gain, with a 45.2 percentage point increase in Afghanistan through a community dialogue intervention [39], while Habib et al. documented an increase from 53.6% to 77.4% in Pakistan via a holistic mother-and-child healthcare programme [32]. Mechael et al., also in Pakistan, reported a reduction in ZD children (no Penta1) from 36.3% to 28.5% over five years using a digital immunization registry, corresponding to a 7.8% increase in Penta1 uptake [47]. In Kenya, Shikuku et al. reported a modest improvement in Penta1 coverage from 96.2% to 102% at six weeks of age through door-to-door outreach; the study commented that the demographic data may not be very accurate; hence performance as per the results may be over 100% [50]. Hamman et al. reported variable post-intervention Penta1 coverage across local government areas in Nigeria, ranging from 0.2% to 16%; *p*-values for these comparisons were not reported [43] (see Table 4). In Pakistan, Penta1 coverage went up by 23.8 percentage points [7].

In addition to the five studies reporting Penta1 outcomes, five studies further reported on OPV0, mostly set within polio-specific programming, and reported changes in OPV0 coverage. The most substantial gains were reported by Khan et al. in conflict-affected Pakistan, where children who had not received OPV0 decreased dramatically from 31.6% to 0.9% through an integrated multi-component intervention [7]. Ongwae et al. reported a tenfold increase in children receiving OPV0 in Nigeria through mobile teams and community mobilizers, while Tessema et al. showed that sustained outreach in Ethiopian pastoralist communities yielded OPV0 coverage two to three times higher than regional averages [5,44]. Nkwogu et al. reported a modest reduction in children who had not received OPV0 from 1.7% to 1.1% in conflict-affected Borno State, Nigeria [6]. As OPV0 is not part of the standard Gavi/WHO zero-dose indicator, these findings are reported separately and should be interpreted as reflecting polio programme reach rather than zero-dose status.

Overall, the largest reported increases in vaccine uptake among previously unvaccinated children were observed in studies combining outreach, community engagement and health workforce strategies (Table 4).

## 4. Discussion

The findings of this scoping review underscore the diversity of strategies implemented to reach ZD and under-immunized children across varying economic and geographic contexts with reported improvements in vaccine coverage across most included studies. The predominance of studies from low- and lower-middle-income countries aligns with the review’s objective to map interventions where the burden of ZD children is highest, with two-thirds of ZD children living below the international poverty line [1,23,36].

Mobile outreach programmes and engagement with religious and community leaders and community healthcare workers were the most frequently reported approaches for addressing demand- and supply-side barriers [23,36]. The most reported barriers to vaccination services for ZD children concerned the intent to vaccinate, followed by community access, reflecting gaps related to behavioural and social drivers of vaccination and the interaction between health systems and communities [15]. These findings highlight the importance of culturally sensitive and community-centred strategies, which are especially vital in hard-to-reach, underserved populations and sometimes conflict-afflicted areas [7,39]. Community engagement interventions can be organized into thematic areas, including leveraging slum health committees for urban slums or integrating with existing humanitarian response services for conflict settings [12].

The incorporation of technology, such as mobile applications and electronic reminder systems, further illustrates evolving innovative approaches that enhance immunization coverage and adherence, particularly in settings with increasing digital access, from LMICs to HICs [48]. Digital health solutions, including mobile health apps, electronic health records, and data analytics platforms, have demonstrated effectiveness in addressing challenges such as vaccine hesitancy, logistics, and real-time monitoring of vaccine distribution [51]. Mobile health applications with features such as GPS tracking of vaccinator outreach visits, digital records of immunization volume, and identification of children who missed vaccination have been piloted in rural districts with poor immunization coverage [52]. Artificial intelligence-driven chatbots, mobile applications, and social listening tools address vaccine hesitancy by providing tailored communication and combating misinformation, while geographic information systems and AI-based behavioural analysis may improve outreach to underserved populations [53]. However, the economic impact of digital health solutions depends on factors such as infrastructure, implementation, and the extent to which these technologies are integrated into existing healthcare systems [54], requiring careful planning and investment to realize their full potential.

Importantly, the review also reveals variation in intervention types based on country income classification, reflecting differing health system capacities and resource availabilities. Implementation research strategies for immunization aligned primarily with operational primary healthcare levers, particularly monitoring and evaluation, workforce development, and models of care, with digital technologies increasingly deployed for real-time tracking and oversight [16]. Campaign-based and community engagement strategies dominate in low-income countries, addressing immediate barriers through trusted local influencers and direct outreach [36,44]. Meanwhile, lower-middle- and upper-middle-income countries increasingly integrate technology and health system strengthening efforts, tailoring interventions to their evolving epidemiological and infrastructural contexts [47,48]. Tools such as “My Village My Home” used in remote areas to record births and vaccination dates help community-level workers visually assess vaccination status and identify unvaccinated children, while similar paper-based systems like “family folders” in Ethiopia provide affordable alternatives for tracking healthcare services [12].

The improvements in vaccine coverage reported in studies from Afghanistan, Nigeria, Thailand, and Pakistan provide supporting evidence for the potential of multi-faceted approaches, though the diverse study designs limit the strength of the evidence [24,35,39]. However, it is important to acknowledge that many of the observed gains were measured over relatively short follow-up periods, raising questions about the sustainability of these improvements beyond the intervention window. Geographic targeting of resources to support expansion of routine immunization services remains critical, though understanding the social, political, and economic contexts of ZD and under-immunized children is equally important for programme design, as factors beyond geography may be more determinative of vaccination status [1]. Together, these findings provide a comprehensive mapping of strategies that can inform policymakers and programme implementers aiming to close immunization gaps and ultimately reduce the global burden of ZD and under-immunized children.

The overall evidence base underpinning this review remains limited. Only 37 studies met the inclusion criteria; therefore, conclusions about the generalizability of individual strategies, particularly for ZD populations, should be interpreted with this small sample size in mind. Most of the studies included in this scoping review are from low- and low–middle-income countries, which limit how far findings can be assumed or transferrable to upper-middle- and high-income countries where barriers to immunization may differ substantially. The review also points to major gaps in the literature, including sparse geographic representation outside sub-Saharan Africa and South Asia.

Another notable limitation in this review is the scarcity of studies that specifically reported outcomes on ZD children as defined by Gavi (i.e., children who have not received the first dose of DTP). A recent scoping review found no studies on interventions that focused specifically on reaching ZD children with routine immunization services, highlighting a critical evidence gap [15]. Most included studies focused on under-immunized children, those who had missed some or latter doses of vaccines such as polio, measles, DTP3, BCG, or Hepatitis B (HepB).

Moreover, many studies referred to under-immunized children as ZD children, which introduces ambiguity and challenges in distinguishing between these two groups. This pattern reflects a broader definitional inconsistency documented in the zero-dose literature. Wonodi and Farrenkopf’s comparative analysis found that using a single-antigen proxy (DTP1/Penta1) versus a composite ‘truly zero-dose’ indicator meaningfully changes which children are classified as zero-dose, with nearly half of ‘penta-zero-dose’ children having received at least one other antigen [4]. The OPV0-specific studies identified in this review did not apply this composite approach, but used OPV0 alone within polio-programme contexts, further illustrating the range of indicator choices used across the primary literature and reinforcing the need for standardized zero-dose reporting.

This lack of clear differentiation limits our ability to fully analyze and discuss interventions targeted exclusively at ZD children versus those aimed at under-immunized populations. This distinction is consequential, as ZD children and under-immunized children represent fundamentally different populations with distinct barriers to vaccine access and uptake. Under-immunized children have had at least some prior contact with immunization services, suggesting that demand exists and that logistical or system-level factors may account for incomplete coverage; interventions such as reminder systems, defaulter tracing, or schedule optimization may therefore be sufficient to improve their immunization status.

ZD children, by contrast, have had no contact with immunization services, often reflecting deeper and more entrenched barriers including geographic isolation, caregiver mistrust, vaccine hesitancy, socio-economic marginalization, conflict, displacement, or systemic exclusion from health services. Interventions designed primarily to improve adherence or reduce dropout among partially immunized children may therefore be insufficient or poorly suited to the needs of ZD populations, who may first require sustained trust-building, community engagement, and targeted outreach before accessing vaccination services. Applying a one-size-fits-all approach risks overstating the effectiveness of interventions for the hardest-to-reach children and diverting attention from the more intensive, context-sensitive strategies that ZD populations require [16]. Future research should prioritize delineating the lived experiences of ZD and under-immunized children and evaluate mitigation strategies specifically addressing equitable vaccine access for ZD children.

Lastly, this scoping review was limited to the peer-reviewed literature (primary research). Grey literature sources, including reports from ministries of health, international organizations, and non-governmental organizations, were not included. This may have introduced publication bias and could mean that programmatic evidence on strategies to reach zero-dose and under-immunized children implemented outside of academic research settings was not captured. Future scoping reviews on this topic would benefit from including grey literature sources to provide a more comprehensive evidence base.

Furthermore, variability in the rigour of study designs and data sources across included studies, ranging from pre–post assessments without comparison groups to studies relying on self-reported or administratively recorded vaccination data, limits the strength of causal inferences. Because no formal critical appraisal was conducted to keep with the standard scoping review methodology, we were unable to weigh or filter findings by study quality. This variability in study rigour should be understood as a constraint on the strength of the conclusions drawn across this evidence base, rather than as a limitation of any single study.

## 5. Conclusions

The blurred distinction between ZD and under-immunized children in current studies highlights the need for clearer definitions and standardized reporting to better guide interventions. The findings emphasize the need for interventions that address the root causes of low uptake of vaccines and other essential services, privileging community-centric approaches [15]. Studies to test and publish interventions designed to identify and reach unimmunized or ZD children should be prioritized.

Practitioners and policymakers must prioritize accurate identification of ZD children to tailor strategies that effectively reach those who have never been vaccinated. ZD children and their families face multiple deprivations related to wealth, education, water and sanitation, nutrition, and access to other health services, and they account for one-third of all child deaths in low- and middle-income countries [1], making it critical to address these interconnected vulnerabilities. Future research should focus on disaggregating data and evaluating interventions specifically targeting real ZD populations. Addressing this gap will be critical for designing evidence-based policies and programmes that close immunization coverage disparities globally.

## Figures and Tables

**Figure 1:**
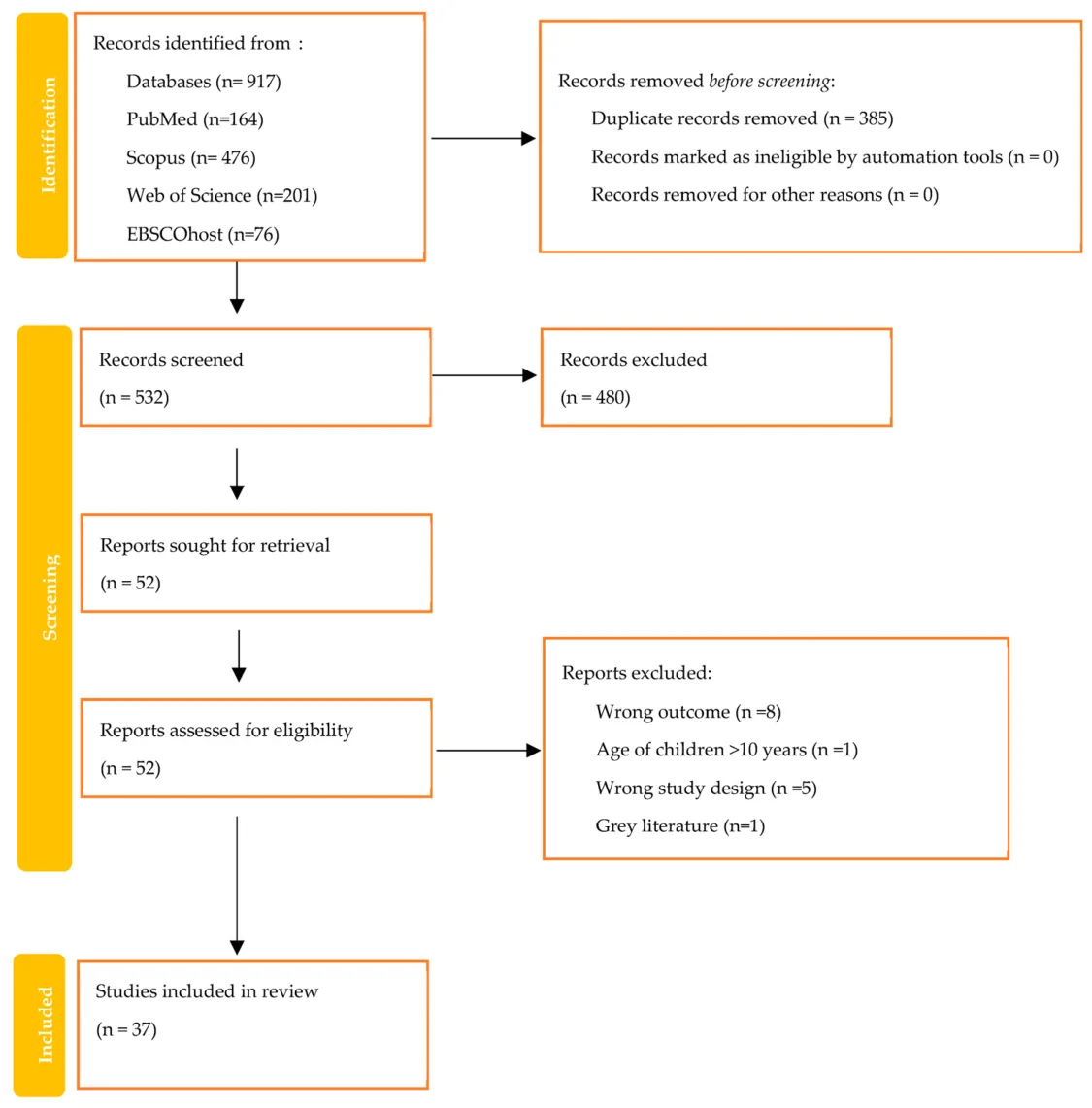
PRISMA flow diagram showing the identification, screening and inclusion of studies into this scoping review. Adapted from [22].

**Figure 2:**
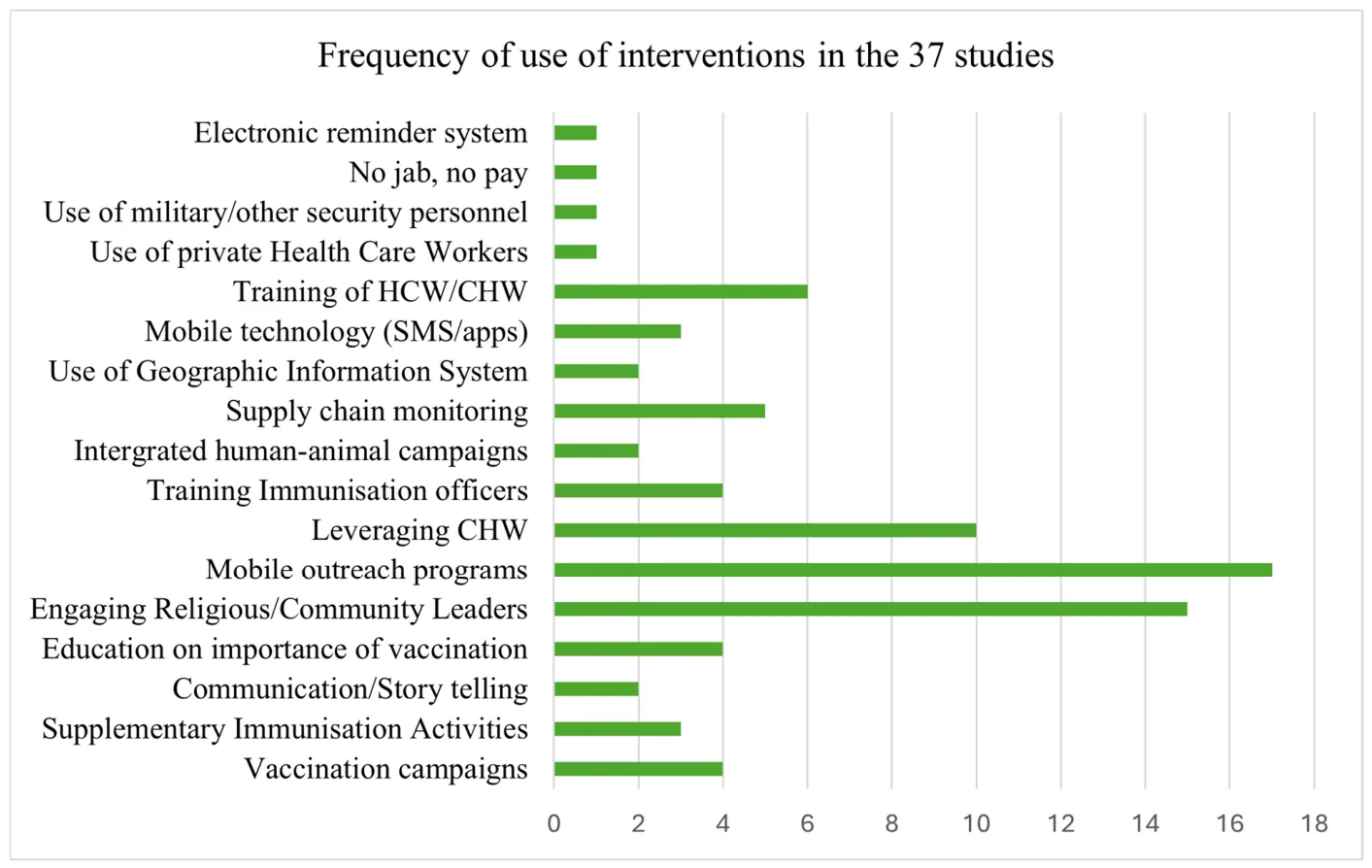
Frequencies of interventions aimed at reaching zero-dose and under-immunized children across 37 studies.

**Table 1:** The search strategy in PubMed.

Search	Query
#1	“Infant”[Mesh]) OR “Infant, Newborn”[Mesh]) OR (“Child”[Mesh] OR “Child, Preschool”[Mesh])) OR “Pediatrics”[Mesh]) AND ((“Immunization”[Mesh] OR “Vaccination”[Mesh]) OR “Vaccines”[Mesh]))
#2	(zero-dose [Title/Abstract] OR “zero dose”[Title/Abstract] OR unvaccinated [Title/Abstract] OR “under vaccinated”[Title/Abstract] OR non-immunized [Title/Abstract] OR hard-to-reach [Title/Abstract] OR “missed communities”
#3	“Strategies [Title/Abstract] OR interventions [Title/Abstract] OR innovations [Title/Abstract]))
#4	#1 AND #2 AND #3
#5	(COVID-19[Title/Abstract] OR COVID [Title/Abstract] OR “COVID 19”[Title/Abstract] OR “SARS-CoV-2”[Title/Abstract] OR hpv[Title/Abstract] OR “human papillomavirus”[Title/Abstract] OR flu [Title/Abstract] OR influenza [Title/Abstract])
#6	#4 NOT #5
Filters	Limit to 2015–2025 (year of publication), <5 years (age)

**Table 2:** Thematic areas of interventions and strategies by income classification.

Thematic Areas	6 LIC	8 LMIC	2 UMIC	2 HIC
Community Engagement	3 (50%)	6 (75%)	2 (100%)	0 (0%)
Community HCWs And Health Workforce	5 (83%)	5 (63%)	1 (50%)	0 (0%)
Health Systems Strengthening	2 (33%)	3 (38%)	0 (0%)	0 (0%)
Integrated Human–Animal Campaigns	2 (33%)	0 (0%)	0 (0%)	0 (0%)
Outreach And Service Delivery	3 (50%)	7 (88%)	1 (50%)	0 (0%)
Technology-Driven	0 (0%)	2 (25%)	1 (50%)	1 (50%)
Leveraging Military And Security Forces	0 (0%)	2 (25%)	0 (0%)	0 (0%)
Monetary Incentives	0 (0%)	0 (0%)	0 (0%)	1 (50%)

Countries by income classification (World Bank, FY2025): LICs = 6; Afghanistan, Chad, Ethiopia, Malawi, Somalia, and Uganda. LMICs = 8; Angola, Bangladesh, Cameroon, Côte d’Ivoire, India, Kenya, Nigeria, and Pakistan. UMICs = 2; China and Thailand. HICs = 2; Australia and Denmark. LIC: Low-income country, LMIC: low–middle-income country, UMIC: upper-middle-income country, HIC: high-income country, CHW: community healthcare worker.

**Table 3:** Summary of intervention themes, settings, and outcomes reported across included studies.

Thematic Area	Description of Intervention	Setting(s) Reported	Outcome(s) Reported	Countries Where Theme is a Standalone Intervention	Countries Where Theme is Embedded in Combined Interventions	Notes
Community engagement	Community sensitisation, engagement with local leaders, caregiver education, and social mobilization activities implemented to improve acceptance and demand for immunization.	Rural; underserved; hard-to-reach; conflict-affected; pastoralist/nomadic	Improved caregiver awareness and acceptability of immunization; increased immunization coverage (within combined interventions).	None identified	Nigeria ([5,6,23,24,25]; Cameroon [26]; Angola [27]; Ethiopia [28]; Somalia [29,30]; Pakistan [7,31,32,33]; India [34]; Thailand [35]; Chad [36]; Cote d’Ivoire [37]; Malawi [38]; Afghanistan [39]; Uganda [40]; China [41]	Reported only as a supporting component within multi-component interventions; rarely evaluated independently.
CHW/HCW	Deployment and training of community and healthcare workers to identify ZD or under-immunized children, deliver vaccines, and conduct follow-up and defaulter tracing.	Rural; underserved; remote; hard-to-reach; fragile settings	Increased immunization coverage; improved identification and follow-up of zero-dose or under-immunized children; improved continuity of immunization services.	Uganda [40]	Angola [27]; Cameroon [26]; Nigeria [5,23,24,25,42,43]; Ethiopia [28,44]; Somalia [29,30]; Afghanistan [39]; Pakistan [7,32,33]; Chad [36]; Cote d’Ivoire [37]	Core delivery strategy; frequently combined with outreach and health systems strengthening.
Outreach and service delivery	Mobile clinics, outreach vaccination sessions, and periodic intensification of routine immunization services targeting underserved populations (campaigns, SIA).	Rural; remote; hard-to-reach; conflict-affected; border areas	Improved access to immunization services; increased number of children immunized; increased immunization coverage among previously unreached populations.	None	Angola [27,45]; Nigeria [5,6,10,23,24,25,43,46]; Cameroon [26]; Pakistan [7,31,32,33]; Somalia [29,30]; Ethiopia [28,44]	Often delivered alongside CHWs, technology-driven tools, or security support.
Technology-driven interventions	Use of mobile health applications, SMS reminders, GIS mapping, and digital tracking tools to support identification, monitoring, and follow-up of ZD or under-immunized children.	Urban; rural; remote; hard-to-reach; conflict-affected; nomadic	Improved identification of zero-dose or under-immunized children; improved timeliness of immunization; increased immunization coverage; improved immunization microplanning.	Pakistan [47]; Bangladesh [48]; Denmark [49]	Pakistan [33]; Thailand [35]	Technology acts as a delivery enabler and is frequently embedded within broader intervention packages.
Leveraging military and security forces	Use of military or civilian security personnel to facilitate access, logistics, and delivery of immunization and child survival interventions, including partnership with armed forces.	Conflict-affected; insecure; hard-to-reach areas	Improved reach of immunization and child survival services to previously inaccessible populations.	Angola [45]	Nigeria [6]	Applied in settings with security constraints to enable outreach and service delivery.
Integrated animal and human vaccination campaigns	Simultaneous delivery of human and animal vaccination services using a One Health approach to reach pastoralist and nomadic populations.	Pastoralist; nomadic; remote rural; hard-to-reach	Increased immunization coverage among children in pastoralist communities; improved access to and delivery efficiency of immunization services for mobile populations.	None	Somalia [29]; Chad [36]	Equity-focused strategy tailored to mobile and pastoralist communities.
Health systems strengthening (HSS)	Interventions aimed at strengthening immunization systems, including supply chains, workforce capacity, governance, and integration within primary healthcare.	Rural and remote health facilities	Sustained increase in vaccine doses delivered; improved immunization system performance and service continuity.	None	Angola [27]; Cameroon [26]; Nigeria [24,25,43]; Pakistan [7,31,47]; Chad [36]; Ethiopia [44]	Commonly implemented as part of multi-component delivery packages rather than standalone strategies.
Monetary incentives	Monetary or benefit-linked incentives encouraging vaccination uptake, including policies linking access to social benefits to complete immunization status.	National/policy-level	Increased immunization coverage; improved timeliness of childhood immunization.	Australia [17]	None	Represented by the “No Pay, No Jab” policy; not combined with programme-level delivery interventions in the included studies.

ZD: Zero-dose children, CHW: community healthcare worker, HCW: healthcare worker.

**Table 4:** Studies reporting on vaccine uptake among ZD (no DTP1/Penta1) children and those with OPV0 coverage.

	Country	Intervention Themes	Duration	Baseline Coverage/Prevalence	Endline Coverage/Prevalence	Change	Age Group
DTP1/PENTA1 COVERAGE							
Mechael et al. [47] 2024	Pakistan	Technology-driven (GIS, electronic immunization registry)	2014–2019	36.3% (ZD children aged 6–23 months, 2014)	28.5% (ZD children aged 6–23 months, 2019)	7.8 pp decrease in ZD children	6–23 months
Hemat et al. [39] 2023	Afghanistan	Community engagement; community and healthcare worker deployment	Oct 2018–Nov 2019	Not reported	Not reported	45.2 pp increase in immunization (intervention arm)	24–35 months
Shikuku et al. [50] 2019	Kenya	Outreach and service delivery	Jan 2017–Jun 2018	96.2%	102%	5.8 pp increase in immunisations	6 weeks
Hamman et al. [43] 2023	Nigeria	Community engagement; community and healthcare worker deployment; outreach and service delivery	2021–2022	Not reported	1.2–16% (range across 5 LGAs)	Not reported	6 weeks
OPV0 COVERAGE							
Ongwae et al. [5] 2017	Nigeria	Community engagement; outreach and service delivery	2014–2015	15,541 children (Nov 2013)	158,224 children (Oct 2015)	+142,683 children immunized (+918%)	>12 months
Tessema et al. [44] 2019	Ethiopia	Outreach and service delivery; community and healthcare worker deployment	2013–2017	15–27% (regional values)	49–59% (CORE Group Polio Project areas)	2–3× higher than regional comparator	≤2 weeks
Nkwogu et al. [6] 2018	Nigeria	Military and security force engagement	May–Jul 2016	1.7% ZD (May 2016)	1.1% ZD (Jul 2016)	0.6 pp decrease in ZD children	Not specified
Khan et al. [7] 2024	Pakistan	Outreach and service delivery; community engagement; health systems strengthening; community and healthcare worker deployment	Apr 2021–Apr 2022	No OPV0 during SIA: 31.6%; No routine immunization: 31.9%	No OPV0 during SIA: 0.9%; No routine immunization: 3.4%	No OPV0 during SIA: 30.7 pp decrease; No routine immunization: 28.5 pp decrease	<3 years
PENTA1 AND OPV0 COVERAGE							
Habib et al.[32] 2024	Pakistan	Community engagement; outreach and service delivery; community and healthcare worker deployment	Jun 2013–May 2014	OPV0: 65.9%; Penta1: 53.6%	OPV0: 78.4%; Penta1: 77.4%	OPV0: 12.5 pp increase; Penta1: 23.8 pp increase	<5 years

GIS, Geographic information system; HSS, health systems strengthening; LGA, local government area; OPV0, oral polio vaccine birth dose; Penta1, pentavalent vaccine (first dose); pp, percentage points; SIA, supplementary immunization activity; ZD, zero-dose. ‘Not reported’ indicates that the original study did not provide sufficient data to calculate the metric. Pp: percentage points.

## Data Availability

The original contributions presented in this study are included in the article/Supplementary Material. Further inquiries can be directed to the corresponding author.

## Supplementary Materials

The following supporting information can be downloaded at https://www.mdpi.com/article/10.3390/vaccines14100848/s1, Figure S1: World Bank classification across the 37 studies; Figure S2: Thematic areas identified in the study; Figure S3: World map showing where the studies included in this scoping review were conducted; Table S1: Characteristics of studies included in the scoping review of strategies and interventions to reach zero-dose and under-immunized children (*n* = 37).

## Abbreviations

The following abbreviations are used in this manuscript:

CHW	Community healthcare worker
DPT	Diphtheria, pertussis, and tetanus
GIS	Geographic information system
HCW	Healthcare worker
HSS	Health system strengthening
HIC	High-income country
LGA	Local government area
LIC	Low-income country
LMIC	Low–middle-income country
MeSH	Medical Subject Heading
PRISMA	Preferred Reporting Items for Systematic Reviews and Meta-Analyses
SIA	Supplementary immunization activity
UIC	Upper-income country
ZD	Zero-dose
