# Supplementary material for: Mapping Strategies and Interventions to Reach Zero-Dose and Under-Immunized Children: A Scoping Review
Source: Vaccines (Basel). 2026 Sep 26;14(10):848. doi: 10.3390/vaccines14100848 (PMC13709433; doi:10.3390/vaccines14100848)
Supplement: Supplementary file 1 [file vaccines-14-00848-s001.zip › Supplementary Table S1.pdf]

Mapping of Strategies and Interventions to Reach Zero-Dose and Under-Vaccinated Children

Authors	Year	Title	DOI	Study Type	Aim	Country	Income Classification (World Bank)	Setting	Intervention Type	Description of Intervention	Outcomes	Limitations
O'Neill et al.	2024	Persistence and heterogeneity of the effects of educating mothers to improve child immunisation uptake: Experimental evidence from Uttar Pradesh in India	https://doi.org/10.1016/j.jhealeco.2024.102899	Randomised Controlled Trial (RCT) — follow-up analysis of prior RCT	To evaluate the long-term impact and variability of an educational intervention targeting mothers to improve childhood immunization rates.	India	LMIC	Rural	Educational intervention	Mothers of under-vaccinated children were randomised to receive gain-framed, loss-framed, or no vaccination information via home visits.	DPT3 and measles coverage increased by ~15 and ~22 percentage points respectively, with effects sustained at 30-month follow-up.	Modest sample size and risk of contamination between arms limit subgroup conclusions and policy generalisability.
Hammanyero et al.	2018	Lessons learnt from implementing community engagement interventions in mobile hard-to-reach (HTR) projects in Nigeria, 2014–2015	https://doi.org/10.1186/s12889-018-6193-z	Descriptive retrospective study (program review)	To describe processes and methods for community engagement, evaluate CE structure performance, and assess impact on immunization coverage in northern Nigeria.	Nigeria	LMIC	Rural, hard to reach	Engaging religious/community leaders; Intervention done by HCWs including CHWs	Traditional and religious leaders, VDC members, and town announcers mobilised communities and conducted house-to-house visits to drive demand for mobile outreach immunisation sessions.	Penta3 coverage rose from 22% to 62% and OPV coverage from 44% to 76% within one year.	Community engagement alone cannot be credited for coverage gains, as concurrent incentives and improved service delivery likely contributed.
Utazi et al.	2024	Geospatial Variation in Vaccination Coverage and Zero-Dose Prevalence at the District, Ward and Health Facility Levels Before and After a Measles Vaccination Campaign in Nigeria	10.3390/vaccines12121299	Geospatial analysis / ecological study (cross-sectional post-campaign survey data)	To generate estimates of MCV zero-dose or unvaccinated children within health facility catchment areas and at ward level, before and after the campaign.	Nigeria	LMIC	Hard-to-reach	Supplementary immunisation activities	A measles vaccination campaign across 13 Nigerian states used two-stage cluster sampling and post-campaign surveys to map zero-dose children at ward and facility level.	Despite 79–89% overall campaign coverage in some states, coverage among zero-dose children remained below 60% in several LGAs.	Reliance on caregiver recall and assumptions in health facility catchment delineation constrain precision of findings.
Mechael et al.	2024	Evaluating the 'Zindagi Mehfooz' Electronic Immunization Registry and Suite of Digital Health Interventions to Improve the	doi: 10.2196/52792	Mixed methods study (retrospective data analysis + qualitative interviews)	To evaluate the ZM-EIR suite of digital health interventions to improve immunization outcomes for children aged 12–23 months	Pakistan	LMIC	Hard-to-reach	GIS; Mobile technology	The Zindagi Mehfooz electronic registry used SMS reminders, QR-code child identification, geospatial tracking, and	Zero-dose prevalence dropped from 36.3% to 28.5% between 2014 and 2019, with modest coverage	Attribution to the digital intervention is uncertain, as similar gains were observed in non-intervention provinces with concurrent

Authors	Year	Title	DOI	Study Type	Aim	Country	Income Classification (World Bank)	Setting	Intervention Type	Description of Intervention	Outcomes	Limitations
		Coverage and Timeliness of Immunization Services in Sindh, Pakistan: Mixed Methods Study			in Sindh Province.					mobile data entry to improve immunisation monitoring and coverage.	gains across BCG, Penta, and measles vaccines.	health system strengthening.
Fattorini et al.	2019	Strengthening Routine Immunization Services in an Angolan Comuna: The Fight against the Burden of Unvaccinated Children in the Sustainable Development Goals Era	10.3390/ijerph16224572	Pre-post intervention study (quasi-experimental)	To evaluate the effect of CUAMM's multi-component intervention to strengthen routine immunization services in the Comuna of Mucope.	Angola	LMIC	Remote, hard to reach	Training of CHW/health care workers; Mobile and outreach programs; Engaging religious/community leaders; Supply chain	A multi-component intervention combined staff training, mobile outreach to communities over 5km from vaccination points, CHW and TBA engagement, and participatory outreach planning every four months.	Total vaccine doses administered increased by 26%, driven by a 62% increase in outreach-delivered doses, with significant gains for OPV2, OPV3, Penta3, PCV3, and Rota2.	Reporting bias from cross-border vaccination card use and absence of a secular trend control limit causal attribution.
Sangwe et al.	2024	The use of community-oriented primary care (COPC) model to generate vaccine demand: The case of a remote fishing community in Cameroon	https://doi.org/10.1016/j.vaccine.2024.126173	Programme evaluation / descriptive intervention study	To describe how the COPC model-based CHW service package enhanced vaccine demand generation in hard-to-reach fishing communities.	Cameroon	LMIC	Remote, hard to reach	Training of CHW/health care workers; Mobile and outreach programs; Engaging religious/community leaders; Intervention done by HCWs including CHWs	CHWs using the Community-Oriented Primary Care (COPC) model conducted home visits, screened zero-dose children, and coordinated with community leaders and a mobile medical team for outreach.	Cumulative vaccination coverage reached 64% among zero-dose and under-immunised children under two years, up from less than 10% at baseline.	No control sites and purposive community selection limit the ability to assess impact at scale.
Sauvageot et al.	2017	Oral cholera vaccine coverage in hard-to-reach fishermen communities after two mass campaigns, Malawi, 2016	http://dx.doi.org/10.1016/j.vaccine.2017.07.104	Cross-sectional coverage survey (post-campaign evaluation)	To assess OCV coverage after two successive campaigns among populations within 2km and 25km of Lake Chilwa in Malawi.	Malawi	LIC	Rural	Campaign	Two OCV campaigns around Lake Chilwa used directly observed vaccination for first doses and innovative self-delivery and community-supervised strategies for second doses in remote areas.	Two-dose coverage reached 75.6% in the 2km zone and 92.2% among lake island residents, with simplified delivery strategies achieving notably high uptake.	Loss of immunisation cards and recall bias between campaign and survey likely introduced coverage measurement error.

Authors	Year	Title	DOI	Study Type	Aim	Country	Income Classification (World Bank)	Setting	Intervention Type	Description of Intervention	Outcomes	Limitations
Grandesso et al.	2018	Oral cholera vaccination in hard-to-reach communities, Lake Chilwa, Malawi	doi: http://dx.doi.org/10.2471/BLT.17.206417	Cross-sectional coverage survey (post-campaign evaluation)	To evaluate vaccination coverage, identify reasons for non-vaccination, and assess satisfaction with innovative second-dose delivery strategies in Lake Chilwa, Malawi.	Malawi	LIC	Rural	Campaign	Standard directly observed vaccination was used for dose one, while zip-lock bag self-delivery and community leader distribution replaced direct observation for dose two in hard-to-reach fishing communities.	Two-dose coverage reached 91.1% on islands and 78.8% among Zimbowera fishermen, with high caregiver satisfaction for the novel strategies.	Absence during the campaign was the primary barrier to vaccination, highlighting ongoing access challenges despite innovative delivery.
Scobie et al.	2015	Cluster survey evaluation of a measles vaccination campaign in Jharkhand, India, 2012	DOI:10.1371/journal.pone.0127105	Cross-sectional cluster survey (post-campaign coverage evaluation)	To estimate measles vaccination coverage in Jharkhand state.	India	LMIC	Rural and urban	Campaign	A large-scale measles campaign delivered vaccines across fixed sites, schools, outreach locations, and special mobile teams targeting high-risk groups in 24 districts.	Overall MCV1 campaign coverage reached 81.1%, but was significantly lower among previously unvaccinated children compared to those already reached by routine immunisation.	Low vaccination card retention required reliance on maternal recall, introducing potential inaccuracy in coverage estimates.
Lu et al.	2017	Implementing the communication for development strategy to improve knowledge and coverage of measles vaccination in western Chinese immunization programs: a before-and-after evaluation	DOI 10.1186/s40249-017-0261-y	Before-and-after study (pre-post evaluation, no control group)	To evaluate the association between the C4D strategy and changes in parental knowledge and measles vaccine coverage.	China	UMIC	Rural, remote	Communication strategy; improve healthcare worker capacity	A one-year C4D project used house-to-house communication, mass media, posters, and culturally tailored educational materials to improve caregiver knowledge and vaccine acceptance across nine provinces.	One-dose measles coverage rose from 83.8% to 90.1% and caregiver willingness to vaccinate increased from 51.3% to 67.4%.	Absence of a concurrent control group and possible social desirability bias limit causal conclusions.
Powell-Jackson et al.	2018	Effect and cost-effectiveness of educating mothers about childhood DPT	https://doi.org/10.1371/journal.pmed.1002519	Randomised Controlled Trial (RCT) — individually randomised	To assess the effect of health information on immunisation uptake via an RCT of health	India	LMIC	Rural	Educational intervention	Mothers of under-vaccinated children received gain- or loss-framed	DPT3 coverage increased by 15 percentage points and	Modest sample size limits detection of framing-specific effects and household

Authors	Year	Title	DOI	Study Type	Aim	Country	Income Classification (World Bank)	Setting	Intervention Type	Description of Intervention	Outcomes	Limitations
		vaccination on immunisation uptake, knowledge, and perceptions in Uttar Pradesh, India: A randomised controlled trial			information messages targeting mothers of unvaccinated or incompletely vaccinated children in rural Uttar Pradesh.					face-to-face information about DPT vaccination benefits during home visits.	measles coverage by 22 percentage points compared to the control group.	proximity introduced contamination risk.
Habib et al.	2017	Community engagement and integrated health and polio immunisation campaigns in conflict-affected areas of Pakistan: a cluster randomised controlled trial	DOI: 10.1016/S2214-109X(17)30184-5	Cluster Randomised Controlled Trial (cRCT)	To evaluate the acceptability and effect on immunisation coverage of an integrated community engagement and MCH immunisation strategy in conflict-affected polio-endemic districts of Pakistan.	Pakistan	LMIC	Conflict-affected	Engaging religious/community leaders; Mobile health camps	A three-arm cluster RCT compared routine polio activities alone against enhanced community outreach with mobile health camps, with a third arm additionally offering inactivated polio vaccine.	OPV coverage increased from 75% (control) to 82–84% in intervention arms, with significant improvements across all districts.	Crowd management challenges, cold chain difficulties, and reliance on family-reported coverage data constrain the reliability and replicability of findings.
Hemat et al.	2023	Increase in institutional delivery and child immunisation coverage through an appreciative inquiry-based community dialogue intervention in Afghanistan	https://doi.org/10.1016/j.puhip.2023.100436	Pre-post intervention study with non-intervention comparison district (quasi-experimental)	To evaluate the effectiveness of a community dialogue intervention using an appreciative inquiry approach in improving institutional delivery and child immunisation coverage in Kandahar, Afghanistan.	Afghanistan	LIC	Rural, hard to reach	Engaging religious/community leaders; Communication strategy; Educational intervention; Intervention done by HCWs including CHWs	Community dialogue sessions grounded in appreciative inquiry engaged community members, religious leaders, and health workers to celebrate vaccination achievements and build collective commitment to full immunisation.	Full immunisation coverage rose from 40% to 78.6%, with BCG, Penta1-3, and Measles-1 all showing substantial antigen-specific gains.	The specific component of the community dialogue driving the improvement could not be identified, limiting replication guidance.
Kaduru et al.	2023	Using community theater to improve demand for vaccination services in the Niger Delta Region of Nigeria		Pre-post intervention study (quasi-experimental with midline and endline surveys)	To improve vaccination demand, acceptance, and uptake in Bayelsa and Rivers State through community	Nigeria	LMIC	Rural, hard to reach, conflict-afflicted	Engaging religious/community leaders; Engaging immunisation managers/HS	An 18-month community theatre intervention used co-created, emotionally driven performances incorporating	Fully immunised children increased from 46% at baseline to 84% post-intervention, with rising child health	Purposive site selection, social desirability bias from emotive performances, and limited male participation constrain

Authors	Year	Title	DOI	Study Type	Aim	Country	Income Classification (World Bank)	Setting	Intervention Type	Description of Intervention	Outcomes	Limitations
					theatre for immunisation.					local languages, traditional songs, and caregiver cast members to address vaccination barriers and build demand.	card availability across the study period.	generalisability and sustainability conclusions.
Kamugisha, S. R et al.	2018	A retrospective cross sectional study of the effectiveness of a project in improving infant health in Bwindi, South Western Uganda	https://doi.org/10.3389/fpubh.2018.00290	Retrospective cohort study (analysis of routine programme data)	To assess whether antenatal and postnatal nurse visits combined with CHV household visits influenced child health outcomes or mortality in Kanungu district, Uganda.	Uganda	LIC	Rural, hard to reach	Intervention done by HCWs including CHWs	Seven nurses and 500 community health volunteers conducted coordinated antenatal and postnatal home visits to identify pregnant women, newborns, and under-vaccinated children across 101 villages.	Postnatal nurse visits were significantly associated with higher immunisation completion (aOR 1.55) and improved infant survival, though children in road-inaccessible villages fared worse.	The bundled intervention package could not be disaggregated to identify which specific component drove the immunisation improvements.
Eba et al.	2023	Exploring the dynamics and causes of poor health care utilization and barriers and facilitators to Mobile Health Services (MHS) in the pastoralist regions of Afar and Somali in Ethiopia	https://doi.org/10.1186/s13570-023-00281-9	Qualitative study (key informant interviews and focus group discussions)	To explore the dynamics and causes of poor health care utilization and implementation barriers and facilitators of MHS in the Somali and Afar regions of Ethiopia.	Ethiopia	LIC	Remote	Mobile and outreach programs	Mobile Health Services (MHS) were deployed to deliver essential health services, including immunisation, to remote pastoralist communities with limited facility access.	MHS improved equitable health access, built community trust, and increased health-seeking behaviour, though quantitative immunisation coverage data were not reported.	Programme sustainability is threatened by absent government policy, donor dependency, logistical challenges, and cultural and language barriers.
Fekadu et al.	2016	Reaching the unreached with polio vaccine and other child survival interventions through partnership with military in Angola	http://dx.doi.org/10.1016/j.vaccine.2016.05.069	Programme description / case study (descriptive operational report)	To describe the Angolan Army health service (AAHS) partnership in polio eradication activities and their contribution to national polio	Angola	LMIC	Remote, hard to reach, conflict-afflicted, border crossing points	Mobile and outreach programs	Angolan Army health service teams were integrated into national polio campaigns, using military vehicles and helicopters to reach mountainous, insecure, and	Military vaccination teams vaccinated 250–400 children per day — double the rate of volunteer vaccinators — with fewer missed	Impact could not be statistically isolated due to lack of disaggregated data from military versus non-military covered areas.

Authors	Year	Title	DOI	Study Type	Aim	Country	Income Classification (World Bank)	Setting	Intervention Type	Description of Intervention	Outcomes	Limitations
					vaccination campaign.					border areas with micro-planned vaccination and social mobilisation.	children in military-covered areas.	
Ongwae et al.	2017	Use of Dedicated Mobile Teams and Polio Volunteer Community Mobilizers to Increase Access to Zero-Dose Oral Poliovirus Vaccine and Routine Childhood Immunizations in Settlements at High Risk for Polio Transmission in Northern Nigeria	DOI: 10.1093/infdis/jiw520	Descriptive programme review (narrative analysis of programme reports)	To demonstrate and justify the use of dedicated mobile teams and polio volunteer community mobilizers in improving polio immunization, routine immunization, and PHC services in high-risk communities across six Nigerian states.	Nigeria	LMIC	Rural, hard-to-reach	Mobile and outreach programs with community mobilizers	Dedicated mobile health teams and polio volunteer community mobilisers (VCMs) were deployed to underserved, high-risk settlements to deliver routine immunisation and primary health care alongside polio eradication activities.	Fully immunised children rose from 19% to 55% and RI card availability from 23% to 56%, with VCMs linking over 156,000 infants to routine immunisation services in 2015.	Findings are specific to high-risk polio settlements and rely on government documents, limiting generalisability and data quality assurance.
Kamadjeu et al.	2015	Immunizing nomadic children and livestock – Experience in North East Zone of Somalia	http://dx.doi.org/10.1080/21645515.2015.1038682	Programme description / case study (operational report)	To interrupt wild poliovirus circulation and reduce measles outbreak risk among nomadic and pastoralist communities in Puntland by integrating human and animal vaccination services.	Somalia	LIC	Rural	Mobile and outreach programs; integrated human and animal vaccination campaign	Human vaccination teams were embedded within a 55-day animal vaccination campaign, using local guides and social mobilisers to reach nomadic and pastoralist children with OPV, measles vaccine, ORS, and vitamin A.	26,393 children received OPV — 34% for the first time — and 23,099 were vaccinated against measles, successfully reaching previously unvaccinated nomadic populations.	High operational cost ($6.20 per child), limited health package breadth, and absence of long-term impact data raise sustainability and scalability concerns.
Bawa	2018	Conduct of vaccination in hard-to-reach areas to address potential polio reservoir areas	https://doi.org/10.1186/s12889-018-6194-y	Descriptive programme report / pre-post comparison	To document the process of implementing integrated mobile vaccination in hard-to-reach areas of Nigeria and to assess its impact on	Nigeria	LMIC	Rural, hard-to-reach	Mobile and outreach programs; Community leaders; vaccination supply monitoring; HCW including CHW	Mobile health teams were deployed to 2,311 high-risk settlements using epidemiological micro-planning, community leader engagement, and a tiered	OPV3 coverage among children under one rose from 23% to 61%, and Penta3 from 22% to 55%.	Absence of cost analysis and difficulty attributing polio interruption solely to the intervention limit conclusions about efficiency and impact.

Authors	Year	Title	DOI	Study Type	Aim	Country	Income Classification (World Bank)	Setting	Intervention Type	Description of Intervention	Outcomes	Limitations
					immunization outcomes.					logistics supply chain to deliver integrated vaccination services.		
Kahow et al.	2024	'Health Camp' model: a unique approach for child vaccination in non-state armed actor controlled, inaccessible geographies in Somalia	https://doi.org/10.1080/16549716.2024.2391598	Programme evaluation / descriptive intervention study	To immunize zero-dose children and enhance continuous access to quality immunization within inaccessible geographies across 10 districts in Galmudug and Hirshabelle states in Somalia.	Somalia	LIC	Rural, remote, hard to reach, conflict-afflicted	Mobile and outreach programs; Engaging religious/community leaders; supplies and cold chain; HCW	Mobile health camps operating five consecutive days monthly in conflict-affected districts delivered EPI, maternal health, and nutrition services through partnerships with local organisations and negotiations with non-state actors.	51,168 zero-dose children aged 0–23 months were identified and immunised across 10 districts between March and December 2023.	The study does not specify which vaccines were administered or confirm that 'zero-dose' refers strictly to children with no DPT1 dose.
Habib et al.	2024	A Holistic Strategy of Mother and Child Health Care to Improve the Coverage of Routine and Polio Immunization in Pakistan: Results from a Demonstration Project	10.3390/vaccines12010089	Pre-post intervention study (demonstration project evaluation)	To assess the impact of an integrated community engagement and MCH immunisation campaign strategy on immunization coverage in Pakistan's high-risk union councils.	Pakistan	LMIC	Rural, urban	Mobile and outreach programs; Engaging religious/community leaders; Intervention done by HCWs including CHWs	Mobile MNCH camps combined with community and religious leader engagement, individual male and female group sessions, and advance community announcements to drive vaccination uptake in high-risk union councils.	OPV4 coverage exceeded 70% and IPV coverage increased substantially across all three provinces, with measles two-dose coverage surpassing 50%.	Reliance on caregiver self-report and on-site recorded doses in conflict-affected areas introduces potential coverage estimation bias.
Ngoshe et al.	2022	Conduct of Integrated Health Services in Real Hard-to-Reach/Under-Served Communities to Address Maternal/Child Health Problems, 2016–2018 in Borno State Nigeria	10.21522/TIJPH.2013.10.02.Art006	Programme evaluation / pre-post comparison (descriptive operational report)	To implement and evaluate the delivery of integrated health services to hard-to-reach and underserved communities in Borno State, Nigeria, from 2016 to 2018.	Nigeria	LMIC	Hard-to-reach and underserved	Mobile and outreach programs; Engaging religious/community leaders; Supply chain	Mobile teams spent up to three days in 1,712 isolated communities across 25 LGAs, supported by community leaders and emergency health kits, to deliver integrated maternal and	Vaccine coverage increased dramatically across all antigens — BCG rose from 32% to 84% and Penta3 from 44% to over 100% — between 2016 and 2018.	Difficult terrain, insecurity, and community resistance to relocation constrained service frequency and long-term health system integration.

Authors	Year	Title	DOI	Study Type	Aim	Country	Income Classification (World Bank)	Setting	Intervention Type	Description of Intervention	Outcomes	Limitations
										child health services.		
Chaudhary et al.	2024	Bridging the Vaccination Equity Gap: A Community-Driven Approach to Reduce Vaccine Inequities in Polio High-Risk Areas of Pakistan	10.3390/vaccines12121340	Pre-post intervention study with difference-in-differences analysis (quasi-experimental)	To evaluate the reduction in vaccination coverage disparities across socioeconomic groups by adopting an integrated community-engagement approach combined with MCH services through mobile health camps.	Pakistan	LMIC	Rural and urban	Mobile and outreach programs; GIS; HCW	Trained community mobilisers combined with GIS-guided mobile health camps delivered routine immunisation and MCH services across 146 union councils in three provinces.	Full immunisation coverage rose from 40.1% to 63.5% in children under 24 months, and zero-dose prevalence dropped by 16.1 percentage points.	Coverage estimates relied on caregiver reports, introducing potential bias particularly in conflict-affected areas.
Kaewkungwal et al.	2015	Application of Mobile Technology for Improving Expanded Program on Immunization Among Highland Minority and Stateless Populations in Northern Thailand Border	http://dx.doi.org/10.2196/mhealth.3704	Pre-post intervention study (quasi-experimental programme evaluation)	To assess outcomes of the 'StatelessVac' project in improving EPI coverage and to evaluate maternal attitudes and practices toward immunizing children among ethnic hilltribe and stateless populations.	Thailand	UMIC	Rural, border areas	Mobile technology; community leaders; advocacy and volunteers	The StatelessVac project used tablets with photo, audio, and dialect-specific features to identify and track unregistered children, supported by Village Health Volunteers conducting home visits and BCC activities in local languages.	OPV-DTP coverage among one-year-olds increased from 91.7% to 94.4% and monthly coverage variability reduced, with improvements also seen in older age groups.	Technology acceptance was assessed only through unstructured interviews, and variable signal strength in highland areas posed real-time data transmission challenges.
Uddin et al.	2016	Use of mobile phones for improving vaccination coverage among children living in rural hard-to-reach areas and urban streets of Bangladesh	doi:10.1016/j.vaccine.2015.11.024	Quasi-experimental study (pre-post with non-randomised control areas; difference-in-differences analysis)	To assess the feasibility and effectiveness of a mobile phone-based system for vaccination registry, newborn tracking, and parental reminders to improve child vaccination coverage in Bangladesh.	Bangladesh	LMIC	Rural and urban hard-to-reach	Mobile technology	The mTika mobile application registered pregnant women, sent automated SMS reminders to mothers and health workers, and enabled supervisor monitoring via smartphone and web interfaces in rural and urban	Full vaccination increased from 58.9% to 76.8% in rural intervention areas (DID +29.5%) and from 40.7% to 57.1% in urban areas (DID +27.1%).	Short intervention period, non-randomised design, population mobility, and reliance on maternal recall limit causal inference and generalisability.

Authors	Year	Title	DOI	Study Type	Aim	Country	Income Classification (World Bank)	Setting	Intervention Type	Description of Intervention	Outcomes	Limitations
										hard-to-reach areas.		
Shikuku et al.	2019	Door-to-door immunization strategy for improving access and utilization of immunization services in hard-to-reach areas: a case of Migori County, Kenya	https://doi.org/10.1186/s12889-019-7415-8	Cross-sectional review of routine DHIS2 data (pre-post comparison)	To determine the effect of an enhanced door-to-door immunization strategy on improving immunization coverage in hard-to-reach areas of Migori.	Kenya	LMIC	Rural and urban hard-to-reach	Mobile outreach programs	GIS mapping identified poorly performing wards, and nurses supported by CHVs conducted three days of door-to-door defaulter tracing and household-level vaccination in addition to routine fixed-point services.	Children reached increased from 10,744 to 14,809, with BCG coverage rising from 74.4% to 89.9% and full immunisation from 78.6% to 103.9%.	Small number of link facilities, short comparison period, and DHIS2 data quality concerns limit precision.
Tessema et al.	2019	Improvements in Polio Vaccination Status and Knowledge about Polio Vaccination in the CORE Group Polio Project Implementation Areas in Pastoralist and Semi-Pastoralist Regions in Ethiopia	doi:10.4269/ajtmh.19-0022	Repeated cross-sectional surveys (longitudinal programme evaluation using multiple survey rounds)	To analyze oral polio vaccine (OPV) coverage in CORE Group Polio Project (CGPP) program areas between 2011 and 2017, focusing on factors influencing coverage levels.	Ethiopia	LIC	Rural, remote, hard to reach, conflict-afflicted	Mobile outreach programs; training of HCW; Campaign	The CGPP deployed mobile outreach, health worker training, and vaccination campaigns targeting hard-to-reach pastoralist and semi-pastoralist communities from 2011 to 2017.	Polio vaccination coverage in CGPP areas improved from 39.6% to 72.6%, substantially outpacing national trends from demographic health survey data.	Coverage remained below the 80–90% recommended threshold, and limited birth-dose delivery during home deliveries constrained early uptake.
Khan et al.	2024	A Low-Cost, Integrated Immunization, Health, and Nutrition Intervention in Conflict Settings in Pakistan—The Impact on Zero-Dose Children and Polio Coverage	10.3390/pathogens13030185	Quasi-experimental study (pre-post with difference-in-differences analysis)	To assess the coverage and utilization of the intervention and its impact on immunization coverage including routine vaccinations and OPVs among children under five.	Pakistan	LMIC	Rural, conflict-afflicted	Mobile outreach program; Private HCW; Training of HCW; Supply chain; religious and community leaders	Mobile health teams, trained private providers, and religious and community leader engagement delivered routine immunisation and nutrition counselling in conflict-affected union councils.	Fully immunised children rose from 20.2% to 43.2% and zero-dose prevalence dropped from 46% to 1.1% at endline.	Short intervention duration, COVID-19 disruptions, and insecurity-related service interruptions limit assessment of long-term sustainability.
Nkwogu et al.	2018	Impact of engaging security personnel on	https://doi.org/10.1186/s12889-018-6188-9	Descriptive programme report / interrupted	To describe the processes of engagement of security	Nigeria	LMIC	Conflict-afflicted	Use of Civilian Joint Task Force; Military Services	The Civilian Joint Task Force and Nigerian	10,074 children were vaccinated across 33	Multiple concurrent interventions and high

Authors	Year	Title	DOI	Study Type	Aim	Country	Income Classification (World Bank)	Setting	Intervention Type	Description of Intervention	Outcomes	Limitations
		access and polio immunization outcomes in security-inaccessible areas in Borno state, Nigeria		time series analysis	personnel to access security-compromised areas and the impact on immunization outcomes.					military provided security escorts, crowd control, and logistical support to enable vaccination teams to reach insurgency-affected settlements using a 'hit and run' strategy.	previously inaccessible settlements, and zero-dose OPV prevalence declined from 1.7% to 1.1% over three months.	population movement due to conflict make it difficult to isolate the security engagement's specific contribution.
Choudhary et al.	2021	Effectiveness of a community-level social mobilization intervention in achieving the outcomes of polio vaccination campaigns during the post-polio-endemic period: Evidence from CORE Group polio project in Uttar Pradesh, India	https://doi.org/10.1186/s12889-021-11425-0	Quasi-experimental study (time-series comparison between intervention and non-intervention areas)	To assess the effects of a community-level social mobilization (CLSM) intervention on community engagement and polio SIA campaign performance from March 2012 to September 2017.	India	LMIC	Unclear	Mobile and outreach programs; community engagement in conjunction with SIA	Community Mobilisation Coordinators conducted pre-SIA community engagement, accompanied vaccinators, addressed refusals through local influencers, and followed up with families of unvaccinated children during each supplementary immunisation round.	Without CLSM, modelling estimated a 14.1% decline in children vaccinated at booths and a 7.4% drop in resistant households converting to polio acceptance.	Purposive selection of high-risk intervention areas and absence of early quantitative data limit comparability and causal precision.
Erismann et al.	2021	Effects of a four-year health systems intervention on the use of maternal and infant health services: results from a programme evaluation in two districts of rural Chad	https://doi.org/10.1186/s12889-021-12330-2	Programme evaluation (pre-post cross-sectional surveys, no control group)	To evaluate the associations between the PADS interventions and the use of maternal and infant health services in two supported health districts in rural Chad.	Chad	LIC	Rural, mobile pastoralists	Supply chain; HCW training; Community engagement; integrated human-animal vaccination campaigns	The PADS programme combined infrastructure development, supply chain improvement, health worker training, performance-based financing, community behaviour change, and integrated human-animal vaccination campaigns across two rural districts.	Vaccination coverage among mobile pastoralists rose dramatically from 15% to 84%, while coverage in settled communities increased modestly from 80% to 83%.	No control group and reliance on self-reported data limit ability to draw definitive conclusions about programme effectiveness.

Authors	Year	Title	DOI	Study Type	Aim	Country	Income Classification (World Bank)	Setting	Intervention Type	Description of Intervention	Outcomes	Limitations
Beard et al.	2025	Impact of Australia's No Jab, No Pay policy on vaccination uptake – a before-after study in two national birth cohorts	https://doi.org/10.1016/j.lanwpc.2024.101259	Before-after study using two national birth cohorts (quasi-experimental, register-based)	To examine the No Jab, No Pay policy impact on vaccine uptake in children.	Australia	HIC	Not indicated	Payment system	Australia's No Jab, No Pay policy removed childcare benefit entitlements for families with registered vaccination objections, creating a financial incentive to vaccinate.	Full vaccination among zero-dose children with registered objections rose from 1.2% to 14.6%, and zero-dose prevalence nationally declined from 3.0% to 2.6%.	Annual eligibility assessment algorithms, incomplete objection registration, and possible emigration among recorded unvaccinated children introduce measurement inaccuracies.
Funk et al.	2025	Effect of a proactive childhood vaccination reminder system on vaccination coverage and uptake in Denmark: A register-based cohort study	https://doi.org/10.1016/j.vaccine.2025.126934	Retrospective register-based cohort study (two birth cohort comparison)	To assess the effect of introducing a vaccination reminder system on the coverage and uptake of the first four childhood vaccinations.	Denmark	HIC	Rural areas	Electronic Reminder system	An electronic reminder system sent parents invitations two weeks before scheduled vaccinations and follow-up reminders four weeks after missed doses via Denmark's national digital mailbox.	The proportion of unvaccinated children fell from 3.4% to 1.3%, and completion of all three DTaP-IPV/Hib doses improved from 79.0% to 88.3%.	Unmeasured confounders between cohorts and COVID-19 pandemic timing affect comparability, and the study could not verify whether reminder letters were read.
Prosperi et al.	2023	Added value of the measles-rubella supplementary immunization activity in reaching unvaccinated and under-vaccinated children, a cross-sectional study in five Indian districts, 2018–20	10.1016/j.vaccine.2022.11.010	Cross-sectional serosurvey study (post-SIA serological evaluation)	To assess the added value of the 2017–2019 MR Supplementary Immunization Activity in India by evaluating its effectiveness in reaching unvaccinated or under-vaccinated children with MCV.	India	LMIC	Rural, urban slum and urban	Supplementary immunisation activities	A nationwide MR supplementary immunisation activity was evaluated across five diverse Indian districts using cross-sectional serosurveys to assess its reach among previously unvaccinated children.	The proportion of measles zero-dose children reached by the SIA ranged from 45.8% to 94.9% across districts, with substantial variation by setting.	Small numbers of zero-dose children pre-SIA in high-coverage districts limited the precision of district-level estimates.
Aplogan et al.	2020	Performance of the strategy to catch up with drop out and unvaccinated children in Cote d'Ivoire in 2018	10.3166/bspe-2020-0141	Programme evaluation / descriptive study (non-random sampling)	To describe the results and cost of a strategy to reduce by at least 40% the number of children lost to follow-up and unvaccinated.	Cote d'Ivoire	LMIC	Rural and urban	Catch-up strategy targeting drop-out and unvaccinated children	Civil society organisations led community sensitisation and CHW home visits to identify dropout and unvaccinated children and refer them to	52.6% of lost-to-follow-up children were located and 46% vaccinated overall, with 82.7% vaccination coverage	Non-random sampling means results reflect areas with higher unvaccinated child burdens and may not be broadly generalisable.

Authors	Year	Title	DOI	Study Type	Aim	Country	Income Classification (World Bank)	Setting	Intervention Type	Description of Intervention	Outcomes	Limitations
										health facilities for catch-up vaccination.	among unvaccinated children who were found.	
Hamman et al.	2023	Evaluation of a Community-based Intervention to Improve Maternal Newborn and Child Health Coverage in the Rural and Hard-to-reach Communities of Adamawa State Nigeria	DOI: 10.21522/TIJPH.2013.11.04.Art018	Programme evaluation / descriptive intervention study (assumed zero baseline)	To evaluate the effectiveness of mobile outreach and health facility outreach programs in improving key MNCH outcomes in rural, underserved, and hard-to-reach communities of Adamawa State, Nigeria.	Nigeria	LMIC	Rural, underserved, hard to reach	Training of CHW; Mobile and outreach programs; Campaign; Intervention done by HCWs including CHWs	Monthly mobile hard-to-reach and health facility outreach teams — each comprising 4–5 health workers and a community mobiliser — delivered integrated MNCH services to rural underserved communities.	Vaccine coverage varied from 0.2% to 16% across intervention LGAs and antigens, assumed to represent gains from a zero baseline prior to the programme.	The absence of pre-intervention coverage data means impact cannot be formally quantified beyond assumed zero-baseline comparisons.
